# Interleukin-1***β*** Modulates Melatonin Secretion in Ovine Pineal Gland: *Ex Vivo* Study

**DOI:** 10.1155/2015/526464

**Published:** 2015-08-03

**Authors:** A. P. Herman, J. Bochenek, J. Skipor, K. Król, A. Krawczyńska, H. Antushevich, B. Pawlina, E. Marciniak, D. Tomaszewska-Zaremba

**Affiliations:** ^1^The Kielanowski Institute of Animal Physiology and Nutrition, Polish Academy of Sciences, Instytucka 3 Street, 05-110 Jabłonna, Poland; ^2^Institute of Animal Reproduction and Food Research, Polish Academy of Sciences, Tuwima 10 Street, 10-748 Olsztyn, Poland

## Abstract

The study was designed to determine the effect of proinflammatory cytokine, interleukin- (IL-) 1*β*, on melatonin release and expression enzymes essential for this hormone synthesis: arylalkylamine-N-acetyltransferase (AA-NAT) and hydroxyindole-O-methyltransferase (HIOMT) in ovine pineal gland, taking into account the immune status of animals before sacrificing. Ewes were injected by lipopolysaccharide (LPS; 400 ng/kg) or saline, two hours after sunset during short day period (December). Animals were euthanized three hours after the injection. Next, the pineal glands were collected and divided into four explants. The explants were incubated with (1) medium 199 (control explants), (2) norepinephrine (NE; 10 *µ*M), (3) IL-1*β* (75 pg/mL), or (4) NE + IL-1*β*. It was found that IL-1*β* abolished (*P* < 0.05) NE-induced increase in melatonin release. Treatment with IL-1*β* also reduced (*P* < 0.05) expression of AA-NAT enzyme compared to NE-treated explants. There was no effect of NE or IL-1*β* treatment on gene expression of HIOMT; however, the pineal fragments isolated from LPS-treated animals were characterized by elevated (*P* < 0.05) expression of HIOMT mRNA and protein compared to the explants from saline-treated ewes. Our study proves that IL-1*β* suppresses melatonin secretion and its action seems to be targeted on the reduction of pineal AA-NAT protein expression.

## 1. Introduction

The daily changes in environmental light conditions have profound impact on all aspects of vertebrate physiology. A chemical messenger of darkness is melatonin (N-acetyl-5-methoxytryptamine), indoleamine mainly synthesized in the pineal gland. Its production is under photoperiodic control via the suprachiasmatic nucleus, hypothalamic structure located in the anterior hypothalamus above the optic chiasm which generates an endogenous “circadian” rhythm with a period very close to 24 hours. [1, 2]. In all mammalian species, melatonin production is regulated by norepinephrine (NE), which is released from sympathetic nerve fibers exclusively at night [2]. Biosynthesis of melatonin is a well-characterized multistep sequence of reactions starting with the hydroxylation of tryptophan to 5-hydroxytryptophan (5-HTP) by an enzyme tryptophan hydroxylase (TPH). Next, aromatic amino acid decarboxylase (DDC) converts 5-HTP to 5-hydroxytryptamine (serotonin). Then, serotonin is transformed to N-acetylserotonin by arylalkylamine-N-acetyltransferase (AA-NAT). Finally, N-acetylserotonin is converted to melatonin by an enzyme hydroxyindole-O-methyltransferase (HIOMT) [3]. In all vertebrates, the key enzyme in melatonin production is AA-NAT, often called the melatonin rhythm enzyme. It is generally accepted that all mechanisms regulating melatonin synthesis converge at the control of AA-NAT enzyme activity [4]. The melatonin is not stored in the pineal parenchyma but is released directly into the general circulation and into the cerebrospinal fluid immediately after its formation [5]. Thus, the circulating levels of the indoleamine faithfully reflect pineal secretory activity [6]. Plasma level of melatonin increases at night, whereas diurnal levels of these hormone are relatively low [7].

Among numerous actions, melatonin plays a role of immunomodulator, regulating the development, differentiation, and function of lymphoid tissues. Moreover, diurnal and seasonal changes in immune function are thought to directly reflect changes in pineal melatonin production which suggests an important role of circulating melatonin in the development and maintenance of immune function [8]. The melatonin mediated photoperiodic effect on the immune system was evidently demonstrated in the studies performed on hamster. It was shown that the artificial shortening of day lengths or exogenous melatonin injection induced an increase in thymus weight and spleen hypertrophy [9, 10]. The results of other studies also showed that melatonin controls diurnal and seasonal rhythms of leukocyte proliferation [11], cytokine production [12], and NK cell activity in mammalian bone marrow cells. [13]. Melatonin regulates inflammatory and immune processes acting as both an activator and inhibitor of these responses. Melatonin demonstrates endocrine, paracrine, and autocrine effects in the leukocyte compartment and differentially modulates proinflammatory enzymes, controls production of inflammatory mediators such as cytokines and leukotrienes, and regulates the lifespan of leukocytes by interfering with apoptotic processes. Melatonin is a potent antioxidant that allows scavenging of oxidative stress in the inflamed tissues. It can affect lipoxygenase activity, which suggests that melatonin might promote early phases of inflammation on the one hand and contribute to its attenuation on the other hand, in order to avoid complications of chronic inflammation [14].

The process of interaction between the immune system and pineal gland seems to be bidirectional. However, the feedback effect of inflammatory response on the pineal gland neuroendocrine functions is poorly understood. It is well known that inflammatory mediators such as cytokines, prostaglandins, and histamine penetrate the region of brain during an immune/inflammatory challenge [15]. The immune mediators have easy access to the pineal gland because it is part of the brain lacking the blood-brain barrier. A few studies showed that the secretory activity of pinealocytes could be modified by the antigenic stimulation [16], histamine [17], cytokines [18, 19], and prostaglandins [20]. Moreover, it was previously described that the rat pineal astrocytes and microglia react to bacterial endotoxin such as lipopolysaccharide (LPS) and lipoteichoic acid (LTA). The pineal gland reacts to LPS by the existence of membrane receptors for LPS, such as toll-like receptor 4 (TLR4) and cluster of differentiation 14 (CD14) [21]. It was described that the systemic inflammation induced by the injection of LPS stimulated the gene expression of potent and pleiotropic proinflammatory cytokine, interleukin- (IL-) 1*β*, directly in the rat pineal gland [22], which suggests that this cytokine may be involved in the inflammatory-dependent modulation of melatonin secretion.

Therefore, the current study was designed to determine the effect of IL-1*β* on melatonin release and expression of AA-NAT and HIOMT in ovine pineal gland, taking into account the immune status of animals before sacrificing, because our previous study carried out on the pituitary explants showed that immune status of organ donor may affect the responsiveness of the gland on this cytokine action [23].

## 2. Materials and Methods

### 2.1. Animals and Experimental Design

These studies were performed on adult, three-year-old blackface ewes in December during short day period. The animals were maintained indoors in individual pens and were exposed to natural daylight. The ewes were in good condition; that is, their body condition was estimated to be 3 according to a five-point scale [24], and the animals were acclimated to the experimental conditions for one month. The ewes were always within visual contact with other members of the flock to prevent isolation stress. The animals were fed a constant diet of commercial concentrates with hay and water available* ad libitum*. All procedures were performed with the consent of the Local Ethics Committee of Warsaw University of Life Sciences-SGGW.

The animals (*n* = 12) were randomly divided into two groups: control (*n* = 6) and LPS-treated (*n* = 6) ewes. Two hours after sunset the animals were intravenously (i.v.) injected by the appropriate volume of LPS from* E. coli* 055:B5 (400 ng/kg) (Sigma-Aldrich, St. Louis, USA) dissolved in saline (0.9% w/v NaCl) (Baxter, Deerfield, IL, USA) into the jugular vein. The maximum volume of LPS solution (10 mg/L) injected into any animal never exceeded 2.5 mL. The control group received an equivalent volume (based on their body weight) of NaCl. The efficiency of the LPS treatment to induce an inflammatory response in the animal was evaluated through the measurement of the animal's body temperature 1 h before and 3 h after the injection. All procedures of the* in vivo* experiment were performed under the dark using the red light.

### 2.2. Incubation of Pineal Explants

All animals were euthanized by decapitation three hours after the LPS or saline injection. The brain was immediately removed from the skulls, and the pineal gland was dissected and divided into four fragments (explants). The explants were first preincubated for 1 h in 24-well plates (Becton Dickinson Labware, Franklin Lakes, NJ, USA) with medium 199 (M199; 600 *μ*L). The medium was replaced with fresh medium every 15 min. Next, all explants were incubated for additional 30 min in M199. Finally, the explants from each ewe were treated with (1) M199 only (control explants), (2) NE (10 *μ*M; Sigma-Aldrich, St. Louis, USA) (a positive control), (3) IL-1*β* (75 pg/mL; Sigma-Aldrich, St. Louis, USA), or (4) NE + IL-1*β* and incubated for 3 h at 37°C (87% O_2_, 5% CO_2_). The incubation medium consisted of M199 HEPES with Earle's salts, sodium bicarbonate, and HEPES (25 mM) with penicillin-streptomycin (10 mL/L) (Sigma-Aldrich, St. Louis, USA). After 3-hour incubation, the explants were weighed, immediately frozen in liquid nitrogen, and stored at −80°C until assay.

### 2.3. Assays

#### 2.3.1. Melatonin Assay

Melatonin was assayed in the experimental media according to the method of Fraser et al., [25] modified in our laboratory, using ovine antimelatonin serum (AB/S/01, Stockgrand Ltd., Surrey, UK). Synthetic melatonin (Sigma-Aldrich, St. Louis, USA) was used as a standard and [O-methyl-3H]-melatonin (Amersham PLC, Amersham, UK) as a tracer. The sensitivity of the assay was 16.8 ± 8.0 pg/mL and the intra- and interassay coefficients of variation were 10.5 and 13.2%, respectively.

#### 2.3.2. Isolation of mRNA and Protein from Pineal Explants

The total RNA and protein from the explants were isolated using the NucleoSpin RNA/Protein Kit (MACHEREY-NAGEL Gmbh & Co., Düren, Germany). All steps of the isolation were performed according to manufacturer's protocol. The purity and concentration of the isolated RNA were quantified spectrophotometrically. The RNA integrity was confirmed by electrophoresis using 1% agarose gel stained with ethidium bromide.

#### 2.3.3. Real-Time PCR Assay

To synthesize cDNA, the Maxima First Strand cDNA Synthesis Kit for RT-qPCR (Thermo Fisher Scientific, Waltham, USA) and 2 *μ*g of total RNA were used. Real-Time RT-PCR was performed using the HOT FIREPol EvaGreen qPCR Mix Plus (Solis BioDyne, Tartu, Estonia) and HPLC-grade oligonucleotide primers (Genomed, Warszawa, Poland). The primer sequences were designed using Primer 3 software [26, 27] (Table 1). One reaction mixture (total volume: 20 *μ*L) contained 4 *μ*L of PCR Master Mix (5x), 14 *μ*L of RNase-free water, 1 *μ*L of primers (0.5 *μ*L each primer, working concentration 0.25 *μ*M), and 1 *μ*L of the cDNA template. The reactions were run on the Rotor-Gene 6000 instrument (Qiagen, Dusseldorf, Germany). The following protocol was used: 95°C for 15 min and 30 cycles of 95°C for 10 s for denaturation, 60°C for 20 s for annealing, and 72°C for 10 s for extension. A final melting curve analysis was performed to confirm the specificity of the amplification.

The relative gene expression was calculated using the comparative quantification option [28] of the Rotor-Gene 6000 software 1.7 (Qiagen). Four housekeeping genes were examined: glyceraldehyde-3-phosphate dehydrogenase (GAPDH), *β*-actin (ACTB), histone deacetylase 1 (HDAC1), and cyclophilin C (PPIC). Endogenous housekeeping gene was chosen based on the result analysis performed with BestKeeper software [29]. ACTB was selected for normalization of the gene expression of enzymes involved in melatonin synthesis and HDAC1 was chosen for IL-1*β* and IL-1R1. The results are presented in arbitrary units, as the ratio of the target gene expression to the expression of the housekeeping gene.

#### 2.3.4. Poly(A) Tail Length Determination

From the total RNA isolated as described above, mRNAs were isolated using NucleoTrap mRNA (MACHEREY-NAGEL Gmbh & Co., Düren, Germany). The mRNAs were processed using USB Poly(A) Tail Length (Affymetrix, Inc., Cleveland, USA), according to the manufacturer's protocol. In first step, using poly(A) polymerase, a limited number of guanosine and inosine residues were added to the 3′-ends of mRNA. In second step, the tailed-RNAs were converted to cDNA through reverse transcription using the newly added G/I tails as the priming sites. Next, control PCR amplification products were generated using a gene-specific forward and reverse primer set designed upstream of the polyadenylation site (Table 2). The second set of primers uses the gene-specific forward primer and the universal reverse primer provided with the kit to generate a product that includes the poly(A) tails of the gene of interest. The reactions were run on the Tgradient (Biometra GmbH, Goettingen, Germany). The following protocol was used: 94°C for 2 min and 30 cycles of 94°C for 10 s for denaturation, 60°C for 1 min for annealing and extension, after the cycles 72°C for 5 min for final extension, and cooling at 4°C for 1 min. After the PCR, the products were separated on 4% agarose gel stained with ethidium bromide (Sigma-Aldrich, St. Louis, USA) in the presence of ready-to-use DNA M50pz ladder (DNA Gdansk, Gdansk, Poland). The length of poly(A) tails was determined by estimation of the distance between the end of “smear” generated by poly(A) containing PCR products and the minimum expected size of AA-NAT and HIOMT, amplified products: 244 bp (209 bp of AA-NAT 3′ end + 35-bp oligo(dT)-anchor) and 373 bp (338 bp of HIOMT 3′ end + 35-bp oligo(dT)-anchor), respectively.

#### 2.3.5. Western-Blot Assay

Before electrophoresis, the protein concentration in the samples isolated using the NucleoSpin RNA/Protein Kit (MACHEREY-NAGEL Gmbh & Co., Düren, Germany) was quantified by Protein Quantification Assay Kit (MACHEREY-NAGEL Gmbh & Co., Düren, Germany). To the appropriate volume of samples containing 50 *μ*g of total protein the appropriate volume of molecular grade water (Sigma-Aldrich, St. Louis, USA) was added to make the total samples volume of 20 *μ*L. In addition to 20 *μ*L of these samples, 19 *μ*L of Laemmli buffer (Sigma-Aldrich, St. Louis, USA) and 1 *μ*L of *β*-mercaptoethanol (Sigma-Aldrich, St. Louis, USA) were added. The received mixtures were boiled for 3 min. Electrophoresis was performed in the presence of molecular weight marker, Spectra Multicolor Broad Range Protein Ladder (Thermo Fisher Scientific Inc., Rockford, USA). Denatured samples and molecular weight standard were loaded on 4–12% polyacrylamide gels and subjected to electrophoresis in Tris-glycine running buffer in the Protean II xi Cell (Bio-Rad Laboratories, Inc., Hercules, USA), according to the manufacturer's instructions. Next proteins were transferred in Tris-glycine blotting buffer to polyvinylidene difluoride membrane, Immobilon-P (0.45 *μ*m) (Merck KGaA, Darmstadt, Germany), using Trans-Blot SD Semi-Dry Transfer Cell (Bio-Rad Laboratories, Inc., Hercules, USA) for 30 min at 20 V. The membranes were blocked overnight at 4°C in blocking buffer, Tris buffered saline, at pH 7.5 with 0.05% Tween-20 (TBST) (Sigma-Aldrich, St. Louis, USA) containing 3% bovine serum albumin fraction V (Sigma-Aldrich, St. Louis, USA). Next, the membranes were incubated for 1 h with primary rabbit anti-HIOMT polyclonal antibody (cat no. bs-6961R; Bioss Inc., Boston, USA), goat anti-AA-NAT polyclonal antibody (cat no. sc-55612, Santa Cruz Biotechnology Inc., Dallas, USA), and mouse anti-ACTB monoclonal antibody (cat no. sc-47778, Santa Cruz Biotechnology Inc., Dallas, USA) dissolved in blocking buffer in dilution 1–200, 1–200, and 1–1000, respectively. After three-time washing, the membranes were incubated with secondary HRP conjugated antibody: bovine anti-rabbit IgG-HRP (cat no. sc-2379, Santa Cruz Biotechnology Inc., Dallas, USA), donkey anti-goat IgG-HRP (cat no. sc-2304, Santa Cruz Biotechnology Inc., Dallas, USA), and goat anti-mouse IgG1 heavy chain (HRP) (cat no. sc-2304, Abcam, Cambridge, UK) dissolved in blocking buffer in dilution 1–10000. After three-time washing, the visualization of membranes was performed using chromogenic detection by the Pierce 1-Step TMB-Blotting Substrate Solution (Thermo Fisher Scientific, Waltham, USA). After the visualization, the membranes were dried and scanned using EPSON Perfection V370 Photo Scanner (Seiko Epson Corporation, Suwa, Japan). The densitometric analysis of the scanned membrane was performed using ImageJ software (Research Services Branch, National Institute of Mental Health, Bethesda, USA).

### 2.4. Statistical Analysis

The raw data, after passing the normality test, were subjected to repeated-measures two-way analysis of variance (ANOVA, GraphPad Prism, San Diego, CA, USA) followed by a post hoc Sidak's multiple comparison test. Statistical significance was established at *P* < 0.05. Data are presented as normalized to the control of saline-treated group.

## 3. Results

### 3.1. Effect of IL-1*β* on Melatonin Release from the Pineal Explants

**Figure 1:**
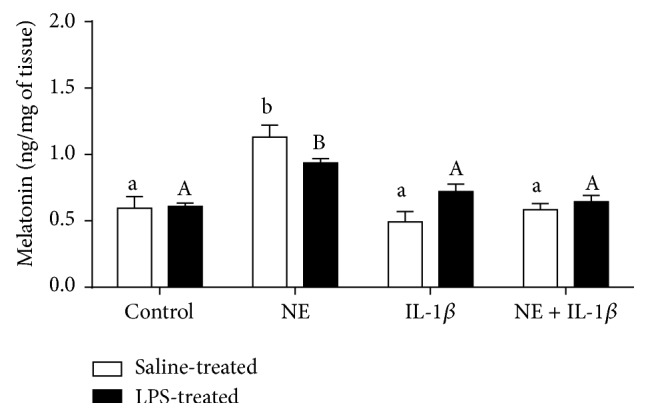
The effects of norepinephrine (NE, 10 *μ*M), interleukin- (IL-) 1*β* (75 pg/mL), and mixture of NE with IL-1*β* on melatonin concentration (pg/mg of tissue) in the medium of pineal gland explants collected from saline- and lipopolysaccharide- (LPS-) treated ewes. Different lowercase letters indicate significant (*P* < 0.05) differences within the saline-treated group; different capital letters indicate differences within the LPS-treated group; control: no NE/IL-1*β* treatment.

Norepinephrine increased (*P* < 0.05) melatonin release from the pineal explants (Figure 1). This effect of NE on melatonin release was completely abolished by IL-1*β*. The explants coincubated with both NE and IL-1*β* did not increase melatonin secretion to the media. There was no influence of animals immune status before sacrificing on the* ex vivo* melatonin secretion.

### 3.2. Influence of IL-1*β* on AA-NAT and HIOMT Expression in the Pineal Explants

**Figure 2:**
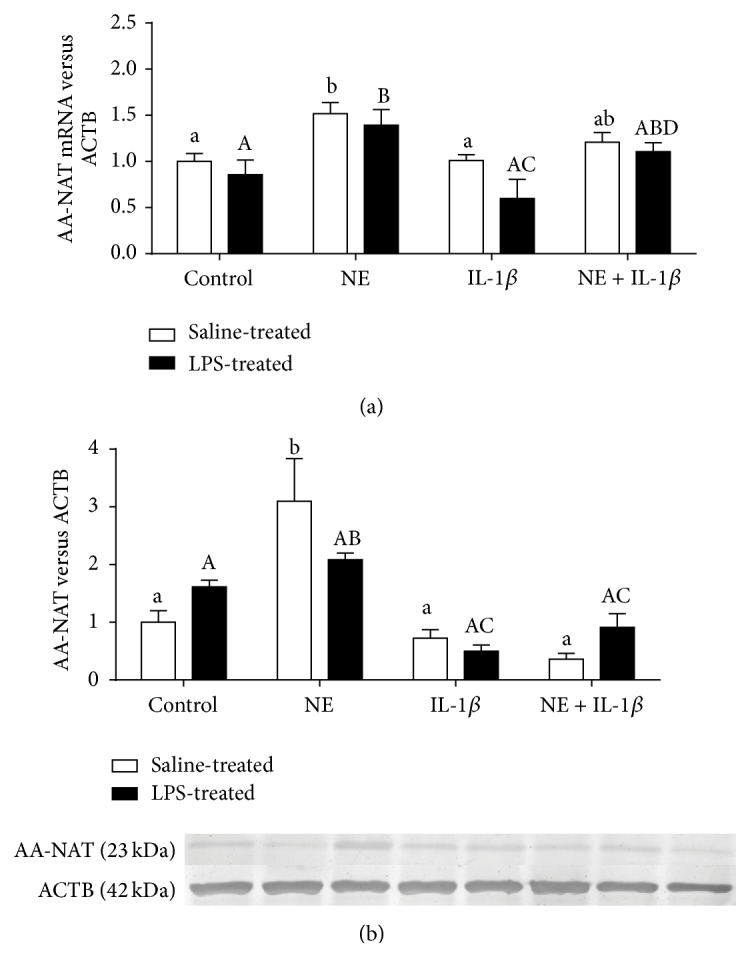
The effects of norepinephrine (NE, 10 *μ*M), interleukin- (IL-) 1*β* (75 pg/mL), and mixture of NE with IL-1*β* on arylalkylamine-N-acetyltransferase (AA-NAT) relative gene (a) and protein (b) expression (mean ± SEM) in the pineal gland explants collected from saline- and lipopolysaccharide- (LPS-) treated ewes. Different lowercase letters indicate significant (*P* < 0.05) differences within the saline-treated group; different capital letters indicate differences within the LPS-treated group.

Norepinephrine administration significantly (*P* < 0.05) stimulated AA-NAT gene expression in the explants from both saline- and LPS-treated ewes (Figure 2(a)). IL-1*β* did not affect basal and NE-stimulated AA-NAT gene expression in the pineal glands from both groups of ewes. Similarly, NE stimulated (*P* < 0.05) significantly AA-NAT protein expression but only in the explants obtained from saline-treated animals (Figure 2(b)). Treatment with IL-1*β* did not affect basal protein expression in both groups; however, it significantly reduced NE-stimulated expression of AA-NAT. There was no effect of LPS treatment on the pineal gland explants response to the treatment.

**Figure 3:**
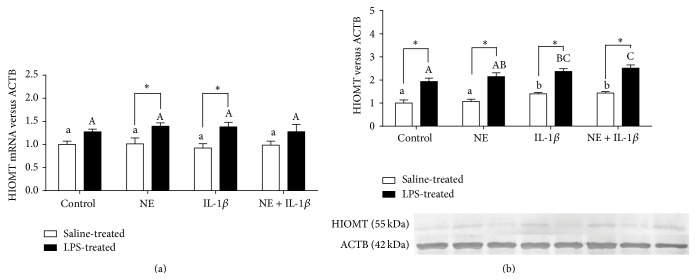
The effects of norepinephrine (NE, 10 *μ*M), interleukin- (IL-) 1*β* (75 pg/mL), and mixture of NE with IL-1*β* on hydroxyindole-O-methyltransferase (HIOMT) relative gene (a) and protein (b) expression in the pineal gland explants collected from saline- and lipopolysaccharide- (LPS-) treated ewes. Different lowercase letters indicate significant (*P* < 0.05) differences within the saline-treated group; different capital letters indicate differences within the LPS-treated group; asterisk designates a significant (*P* < 0.05) difference between the saline- and LPS-treated groups; control: no NE/IL-1*β* treatment.

There was no effect of NE or IL-1*β* treatment on gene expression of HIOMT in the explants from both saline- and LPS-treated ewes. Additionally we observed higher HIOMT mRNA expression in explants collected from LPS-treated than saline-treated animals (Figure 3(a)). Similarly, the pineal explants collected from LPS-treated ewes had significantly (*P* < 0.05) higher expression of HIOMT protein than those from saline-treated ewes. IL-1*β* significantly (*P* < 0.05) increased HIOMT protein expression in the explants from both saline- and LPS-treated ewes (Figure 3(b)). NE did not affect basal and IL-1*β* stimulated protein expression of the pineal glands from both groups of ewes.

### 3.3. Effect of IL-1*β* on the Gene Expression of IL-1*β* and IL-1R1

**Figure 4:**
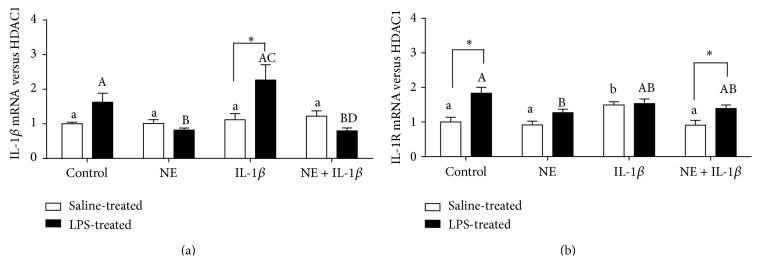
The effects of norepinephrine (NE, 10 *μ*M), interleukin- (IL-) 1*β* (75 pg/mL), and mixture of NE with IL-1*β* on the poly(A) tail length of mRNAs encoding arylalkylamine-N-acetyltransferase (AA-NAT) and hydroxyindole-O-methyltransferase (HIOMT) in the pineal gland explants collected from saline- and lipopolysaccharide- (LPS-) treated ewes. M: DNA ladder M50pz, numbers indicate each experimental group: (1) control, (2) NE, (3) IL-1*β*, and (4) NE + IL-1*β*. The minimum expected size of AA-NAT and HIOMT, amplified products, was 244 bp (209 bp of AA-NAT 3′ end + 35-bp oligo(dT)-anchor) and 373 bp (338 bp of HIOMT 3′ end + 35-bp oligo(dT)-anchor), respectively.

All pineal explants expressed mRNA encoding IL-1*β*. Moreover, in explants obtained from LPS-treated ewes, the gene expression of IL-1*β* was downregulated by NE (Figure 4(a)). On the other hand, IL-1*β* stimulated (*P* < 0.05) the gene expression of its type I receptor only in the explants from saline-treated ewes (Figure 4(b)). However, this stimulatory effect of IL-1*β* on IL-1R1 gene expression was abolished by NE treatment. No influence of IL-1*β* on the IL-1R1 mRNA level was found in explants from LPS-treated animals. In these groups of explants NE reduced IL-1R1 gene expression only when acting alone, because in explants coincubated with NE and IL-1*β* this reducing effect was completely diminished.

### 3.4. Effect of IL-1*β* on the Length of Poly(A) Tail of AA-NAT and HIOMT mRNA

**Figure 5:**
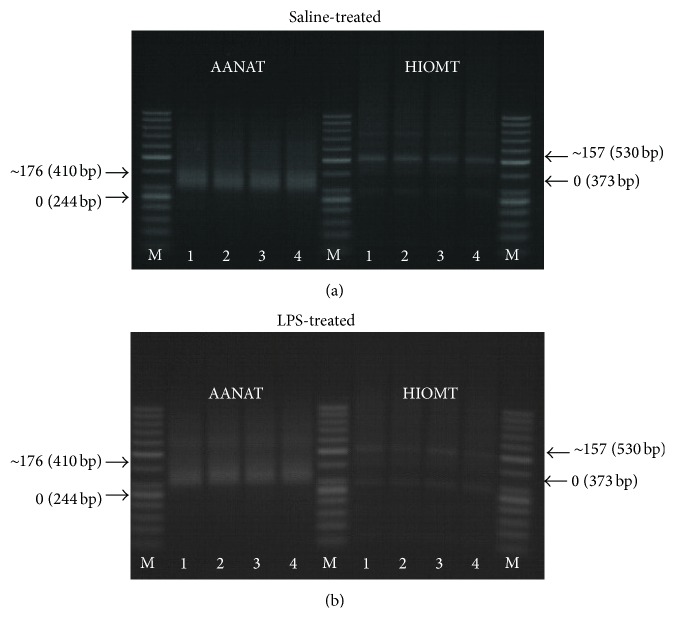
The effects of norepinephrine (NE, 10 *μ*M), interleukin-1*β* (IL-1*β*, 75 pg/mL), and mixture of NE with interleukin- (IL-) 1*β* on IL-1*β* (a) and its type 1 receptor (IL-1R) (b) relative gene expression (mean ± SEM) in the pineal gland explants collected from saline- and lipopolysaccharide- (LPS-) treated ewes. Different lowercase letters indicate significant (*P* < 0.05) differences within the saline-treated group; different capital letters indicate differences within the LPS-treated group; asterisk designates a significant (*P* < 0.05) difference between the saline- and LPS-treated groups; control: no NE/IL-1*β* treatment.

No effects of IL-1*β*, NE, and immune status on the length of poly(A) tail of mRNA encoding enzymes, AA-NAT and HIOMT, were found (Figure 5).

## 4. Discussion

The results of our study demonstrate that IL-1*β* may be an important mediator via immune system which regulates the melatonin secretion. The experiment carried out on ovine pineal explants showed that IL-1*β* suppressed NE-stimulated melatonin secretion. This result fully supports the previously published* in vivo* study displaying the inhibitory action of these cytokines on melatonin secretion in rat [19]. They found that exogenous recombinant human IL-1*β* decreased the melatonin synthesis at dose-dependent manner. Moreover, this effect of IL-1*β* was abolished by introduction of the anti-human IL-1 receptor antibody. Our* ex vivo* study also showed that IL-1*β*-induced decrease in melatonin release generally reflects the changes occurring in AA-NAT protein expression, because the effect of IL-1*β* on the transcription of this enzyme seems to be more ambiguous. It should be pointed out that the regulatory signals that control AA-NAT and melatonin biosynthesis vary among vertebrate species [2]. In all mammals NE regulates the melatonin synthesis by activation of two subtypes of adrenergic receptors. Activation of *β*1-adrenergic receptors increases the intracellular concentration of cAMP followed by activation of the cAMP-dependent protein kinase A (PKA). Both elevated cAMP level and PKA activation are indispensable for stimulation of AA-NAT and melatonin synthesis in all mammalian species. In turn, activation of *α*1-adrenergic receptors increases the intracellular calcium ([Ca^2+^]_*i*_) concentration caused by release of calcium ions from intracellular stores [30]. The NE-dependent activation of the *β*1-adrenergic/cAMP/PKA and *α*1-adrenergic/[Ca^2+^]_*i*_ pathways is conserved in mammalian physiology, but the downstream mechanisms that link these signaling cascades with AA-NAT activation and melatonin production exhibit marked species-to-species variations [2]. In rodents, the cAMP/PKA pathway controls transcriptional mechanisms regulating melatonin synthesis. It was demonstrated that stimulation by NE approximately 100-fold increased cAMP level in the rat pinealocytes [31]. Moreover, the day/night rhythm of changes in cAMP concentration in the rat pineal gland are parallel to the pattern of changes in AA-NAT mRNA expression, which increases by approximately 150-fold during the night [4, 32]. In ungulates and primates, melatonin synthesis is controlled by mechanism targeted on the posttranslational regulation of AA-NAT. In these animals, pinealocytes constantly synthesize AA-NAT protein from continually available AA-NAT mRNA. In the absence of noradrenergic stimulation, AA-NAT protein is destroyed by proteasomal proteolysis. Under NE stimuli, elevated cAMP levels cause phosphorylation of AA-NAT by PKA. This posttranslational modification leads to interaction of phosphorylated AA-NAT with 14-3-3 proteins protecting AA-NAT from degradation [2, 33]. Our results showing that IL-1*β* abolished NE-induced increase in AA-NAT protein expression but did not affect this enzyme transcription suggest that the action of IL-1*β* is targeted on the reduction of the rate of this enzyme proteolysis in the sheep pinealocytes. It is also worth pointing out that in our experiment mild but significant upregulation of AA-NAT gene expression was found in NE-treated explants. This result is consistent with previously published data which demonstrates that in sheep pineal gland slight day/night fluctuation of AA-NAT mRNA level occurs [34]. They found that nocturnal level of mRNA encoding AA-NAT is about 2 times higher than daily level of this transcript. However, the availability of mRNA to translation not only depends on the level of transcription but is also affected by this mRNA stability. The poly(A) tail of mRNA has an important influence on the dynamics of gene expression. On the one hand, it promotes enhanced mRNA stability to allow production of the protein, even after inactivation of transcription. On the other hand, shortening of the poly(A) tail, deadenylation, slows down translation of the mRNA or prevents it entirely, by inducing mRNA decay. Thus deadenylation plays a crucial role in the posttranscriptional regulation of gene expression, deciding the fate of individual mRNAs [35]. Our study also showed that neither NE nor IL-1*β* affects the AA-NAT mRNA stability, because there was no influence of these factors on the length of poly(A) tail of this transcript. This seems to support the thesis that this enzyme expression is mainly regulated at the posttranscriptional level.

Although it is postulated that melatonin secretion is mainly regulated at the AA-NAT level, in our study the expression of HIOMT was also assayed. In contrast to AA-NAT, immune status of animals before sacrificing affected HIOMT expression. The expression of this enzyme was higher in explants collected from LPS- and then saline-treated ewes. However, no effects of experimental treatments were found on HIOMT mRNA poly(A) tail length. The stimulatory effect of inflammatory condition on HIOMT gene expression and HIOMT enzymatic activity was previously described in chicken [3]. Similar to our study, the changes in HIOMT gene expression and activity did not affect melatonin synthesis in these birds, probably due to the simultaneous inhibition of N-acetylserotonin biosynthesis. Due to the lack of data indicating the effect of immune mediators on HIOMT expression and activity based on the studies carried out on rodents, Piesiewicz et al. [3] speculated that these changes could be caused by stress hormones stimulating pineal HIOMT gene expression and activity, which resulted in increased melatonin secretion [36]. However, our findings that IL-1*β* stimulated the protein expression of HIOMT in pineal explants collected from both LPS- and saline-treated animals seem to shed some light on this issue. It suggests that inflammatory-dependent increase in HIOMT synthesis in the pineal gland may be induced by inflammatory mediators such as IL-1*β*.

The synthesis of melatonin may be regulated by circulating IL-1*β*. Our study suggests that IL-1*β* is locally synthesized in the pineal gland; therefore, this cytokine may also affect the melatonin synthesis in paracrine way. We determined relatively high and constitutive IL-1*β* mRNA expression in pineal explants collected from saline-treated ewes. Moreover, in the case of pineal tissues from LPS-treated animals, significant suppression of IL-1*β* transcription was determined under the influence of NE. This result supports previous report about constitutive expression of IL-1*β* in the rat pineal gland, which has also been shown to correlate with the diurnal melatonin rhythm, since IL-1*β* mRNA is higher during the day than during darkness [37]. Unexpectedly, they determined that IL-1*β* expression was upregulated in pineal cultures after treatment with NE. Increased IL-1*β* expression by NE* ex vivo* and the decline in IL-1*β* expression at night when NE levels are elevated were explained based on immunocytochemical data showing that astrocytes are the predominant cell type expressing this cytokine* in vivo*, whereas IL-1*β*-positive cells are predominantly microglia in pineal explants and dispersed cell cultures. They assumed that these two types of IL-1*β* expressing cells may differently affect the pinealocytes activity. However, our study performed on* ex vivo* model suggests that NE rather inhibits than stimulates IL-1*β* expression in pineal gland. It suggests that at least in sheep NE regulates pineal IL-1*β* synthesis via its anti-inflammatory mechanism of action, which was previously reported for other mammalian cells [38, 39].

IL-1*β* may affect the melatonin secretion thanks to the existence of IL-1R1 in the pineal gland. We found that IL-1*β* stimulated the gene expression of its type I receptor, but this effect was also diminished by NE. On the other hand, IL-1*β* did not affect IL-1R1 mRNA expression in the explants collected from LPS-treated ewes but this receptor transcription was also reduced by NE. It is worth mentioning that explants collected from LPS-treated ewes are characterized by higher gene expression of IL-1R1 than those from saline-treated animals. However, without immunohistochemical analysis, it is impossible to judge which type of pineal cells expresses the higher amount of IL-1 receptors. It is postulated that, in addition to direct actions on pinealocytes, cytokines and other immune factors regulate pineal gland functioning by indirect actions on pineal glial cells [8]. It is suggested that the effect of cytokines such as IFN-*γ* and IL-1*β* on pinealocytes may be mediated by microglia, when TNF*α* may exert its biological effects acting directly on pinealocytes [40].

Summarizing, our study supports the thesis that interaction between pineal gland and the immune system is bidirectional. It was found that proinflammatory cytokine, IL-1*β*, suppresses the process of melatonin synthesis in ovine pinealocytes, mainly affecting AA-NAT protein expression. Moreover, the fact that IL-1*β* is synthesized in the pineal gland suggests that not only exogenous but also “local” IL-1*β* may be involved in the regulation of the melatonin synthesis. Our study also shows that immune status of animals affects HIOMT expression in pineal tissue even in* ex vivo* condition, few hours after disappearance of inflammatory stimuli. Although stimulatory influence of both inflammatory condition and IL-1*β* on HIOMT expression is unambiguous, the explanation of physiological significance of this phenomenon requires future detailed studies.

## Figures and Tables

**Table 1:** All genes analyzed by real-time PCR are listed with their full name and abbreviation.

GenBank Acc. number	Gene	Amplicon size [bp]	Forward/reverse	Sequence 5′ → 3′
NM_001034034	GAPDH: glyceraldehyde-3-phosphate dehydrogenase	134	Forward	AGAAGGCTGGGGCTCACT
			Reverse	GGCATTGCTGACAATCTTGA

U39357	ACTB: beta-actin	168	Forward	CTTCCTTCCTGGGCATGG
			Reverse	GGGCAGTGATCTCTTTCTGC

NM_001076910	PPIC: cyclophilin C	131	Forward	ACGGCCAAGGTCTTCTTTG
			Reverse	TATCCTTTCTCTCCCGTTGC

BC108088.1	HDAC1: histone deacetylase 1	115	Forward	CTGGGGACCTACGGGATATT
			Reverse	GACATGACCGGCTTGAAAAT

NM_001009461	AANAT: arylalkylamine-N-acetyltransferase	154	Forward	CGAGAGGCCTTCATCTCTGT
			Reverse	GTCTCTCCTCATCCCACAGG

KC290950	HIOMT: hydroxyindole-O-methyltransferase	167	Forward	AGCTTCCATGAAGGGGATTT
			Reverse	AGGAGGCTCTCGATGACCAG

X54796.1	IL-1*β*: interleukin-1 beta	137	Forward	CAGCCGTGCAGTCAGTAAAA
			Reverse	GAAGCTCATGCAGAACACCA

NM_001206735.1	IL-1R1: interleukin 1 receptor, type I	124	Forward	GGGAAGGGTCCACCTGTAAC
			Reverse	ACAATGCTTTCCCCAACGTA

**Table 2:** Gene-specific primers designed for the poly(A) tail length analysis.

GenBank Acc. number	Gene	Amplicon size [bp]	Forward/reverse	Sequence 5′ → 3′
NM_001009461	AANAT: arylalkylamine-N-acetyltransferase	178	Forward	GAACAGTGACCGCTGACTCC
			Reverse	GCCTCCCCACCTTCTCTTTA

KC290950	HIOMT: hydroxyindole-O-methyltransferase	310	Forward	GTCTTGGCCAGAAAGTGAGC
			Reverse	ACCCAGGAGGAACCTCATCT
